# Relationships Between Vertical Jump Strength Metrics and 5 Meters Sprint Time

**DOI:** 10.2478/v10078-011-0045-6

**Published:** 2011-10-04

**Authors:** Mário C. Marques, Helena Gil, Rui J. Ramos, Aldo M. Costa, Daniel A. Marinho

**Affiliations:** 1 Department of Sports Sciences, University of Beira interior, Covilhã, Portugal; 2 Research Centre for Sport, Health and Human Development, Portugal

**Keywords:** lower extremity, force, power, sprinting

## Abstract

The aim of this study was to examine the relationship between short sprint time (5 m) and strength metrics of the countermovement jump (CMJ) using a linear transducer in a group of trained athletes. Twenty-five male, trained subjects volunteered to participate in the study. Each volunteer performed 3 maximal CMJ trials on a Smith machine. Peak instantaneous power was calculated by the product of velocity taken with the linear transducer. For sprint testing, each subject performed three maximum 5 m sprints. Only the best attempt was considered in both tests. Pearson product–moment correlation coefficients between 5 m sprint performance and strength metrics of the CMJ were generally positive and of clear moderate to strong magnitude (r = −0.664 to −0.801). More noticeable was the significant predictive value of bar displacement time (r= ∼0.70) to sprint performance. Nevertheless, a non-significant predictive value of peak bar velocity and rate of force development measurements was found. These results underline the important relationship between 5 m sprint and maximal lower body strength, as assessed by the force, power and bar velocity displacement. It is suggested that sprinting time performance would benefit from training regimens aimed to improve these performance qualities.

## Introduction

Implementing objective methods to assess physical performance has become an invaluable component of athletic development, monitoring, and talent identification in sport. In terms of lower body exercises, for example, squats and vertical jumps appear to be most widely used to develop sprint performance (González-Badillo and Marques, 2010). Several studies examined the associations between sprint ability and distinct strength and power measures in isoinertial exercises (Young et al., 1995; Marques and González-Badillo, 2006; Harris et al., 2008). Moreover, common motor skills such as sprinting have biomechanical, kinematic, and muscular similarities to vertical jump movement, but determining associations between this task and short sprinting ability has proved elusive (Delecluse et al., 1995; Kukolj et al., 1999; Gorostiaga et al., 2005). Part of these discrepancies could be due to the fact that sprinting is a complex ability (Sleivert and Tringahue, 2004).

Unfortunately, to our best knowledge, few studies have examined the relationship between short sprint performance in trained subjects with indices of dynamic force, impulse, power, and bar velocity during muscle contractions of lower-extremity in the countermovement jump (CMJ). In fact, research has identified that the first few ground contact phases of a short sprint are dominated by propulsive forces and by concentric muscle actions (Mero et al., 1983; Mero, 1988; Habibi et al., 2010). The mechanical impulse of track sprinters in the blocks, for instance, and during the propulsive phase of the first ground contact have also shown significant correlations with initial running velocity. These findings emphasize the dominance of the propulsive phase during initial acceleration, and the importance of propulsive force developed during the first few foot contacts of the sprint in maximizing initial running velocity. Since explosive concentric muscle actions are of major importance to short sprint acceleration (Nesser et al., 1996; Gorostiaga et al., 2005), it seems logical that similar resistance training exercises might be suitable for testing and training these neuromuscular qualities.

According to literature, force platforms would appear to be one of the most commonly used measuring devices in biomechanics (Carlock et al., 2004). However, some problems of using force platforms are the costs and portability due to its weight, which makes it difficult to use in field tests. To avoid these problems a linear transducer could be used since this device can directly measure the position over time. The linear transducer has shown high validity and reliability in its measurements of force when compared to a force platform (Cronin et al., 2004).

None of the previous studies examined short sprinting time (5 m) with dynamic force performance together with power output, mechanical impulse, displacement, time and bar velocity measured with a liner transducer. Therefore, the aim of this research was to examine the relationship between short sprint times (5 m) and strength metrics of the CMJ using a linear transducer in a large data of trained athletes. Examination of these relationships could be of great importance for the optimal development of resistance training programs to improve short sprint performance in athletes.

## Material and Methods

### Approach to the Problem

Twenty-five students were acquainted with all test procedures four weeks before the measurements were applied. All were trained amateur athletes of different sports (e.g. soccer, futsal, and team handball). Consequently, all the participants were well conditioned. Subjects were familiar with all of the testing procedures and exercises, as they had been performing them as part of their regular training routine. The concentric-only portion of the CMJ was taken to analysis.

### Subjects

A group of 25 male trained participants volunteered to participate in the study (mean ± SD: age 21.5 ± 1.3 year, body mass 68.3 ± 5.4 kg, body height 1.74 ± 0.04 m). Before commencing the study, subjects had a physical examination, and each was cleared of any medical disorders that might limit full participation in the investigation. Subjects were required to sign an informed consent form prior to the study that had been approved by the Institutional Review Committee Board of the local Committee for Medical Research Ethics and current Portuguese law and regulations, and was carried out according to the Helsinki Declaration.

### Testing Procedures

Following a standard warm-up, participants performed three maximal CMJ trials in a Smith machine. The bar of this apparatus had a linear transducer attached (Isocontrol, JLML, Madrid, Spain). The rotary encoder of the linear transducer recorded the position and direction of the bar (17 kg) to within an accuracy of 0.0002 m. Peak instantaneous power was calculated by the product of velocity taken with the linear transducer. Each subject initiated the CMJ from a standing position, performed a crouching action followed immediately by a jump for maximal height. Hands remained on the bar for the entire movement in order to maintain contact between the bar and shoulders. Three minutes of rest were provided between each trial to minimize fatigue. The trial-to-trial reliability of the CMJ measured by the linear transducer gave an intraclass correlation coefficient (ICC) of 0.89–0.95 for concentric force, maximum power and maximum rate of force development. The coefficients of variation (CV) were 4–14% with the linear transducer. Only the best attempt was taken for analysis. For sprint testing subjects were required to perform three maximum effort sprints of 5 metres. Times were recorded using Brower equipment (Wireless Sprint System, USA). Subjects performed the sprints with 3 min rest periods. Only the best attempt was considered. The sprints reported an ICC of 0.89–0.96 and CV of 1.8%.

### Statistical Analyses

Mean (± SD) were calculated for each variable. The Kolmogorov-Smirnov test of normality and Levine's test of homogeneity of variance were performed to verify the normality of the distribution. The intraclass correlation coefficient (ICC) was used to determine between-subject reliability of jumping tests. Within-subject variation for all tests was determined by calculating the coefficient of variation (CV) as outlined by Hopkins (2000). Pearson product-moment correlation coefficient was used to verify the association between variables. Data was analyzed using SPSS 12.0 (Lead Tools, 2003). The level of significance was set at p ≤ 0.05.

## Results

Pearson product–moment correlation coefficients between 5 m sprint performance and strength metrics of the CMJ are presented in Table 1. In brief, values were generally positive and of clear moderate to strong magnitude (r = −0.664 to −0.801). More noticeable was the significant predictive value of bar displacement time (r= ∼0.70) to sprint performance. Nevertheless, a non-significant predictive value of peak bar velocity and rate of force development measurements was found.

## Discussion

The purpose of this study was to investigate the relationships between short sprint ability and strength variables during a vertical jump in a group of trained subjects. To our best knowledge, this is the first study attempting to examine this issue with so much extent strength metrics measured with a linear transducer that can better explain short sprint performance in a group of trained athletes as the one presented here. The major findings of this study were the significant correlations between bar displacement/time, peak bar velocity, mean propulsive force, and mean propulsive power measures and sprinting time.

No previous studies were found that reported relationships between bar velocity during a loaded vertical jump and sprint start performance. Gorostiaga et al. (2005) observed a significant relationship between bar velocity during a bench press test using 30% of maximal load and standing ball throwing velocity for elite (r =0.67) and amateur team handball players (r = 0.71). This value is very similar to the one that was found in the present investigation. Taken together these data suggest that both sprinting is related to the capacity to move low loads with lower limbs at maximal velocities. The data observed in this study showed that concentric displacement and times were moderately related with 5 m sprint performance. In contrast, the mean propulsive velocity and peak velocity failed to be significantly associated with 5 meter sprint ability. No study prior to ours attempted to examine these associations, except Sleivert and Taingahue (2004), but only for bar velocity. Conversely to our results, these authors observed a poor but significant correlation (r= − 0.45, p<0.05) between bar velocity and 5 meter performance. However, these differences can be partially explained regarding two points. First, the peak bar velocity used by Sleivert and Taingahue (2004) corresponded to 30% of one maximum repetition during a traditional squat and not free jumping movement as the one presented here. Secondly, the current study used trained students and not elite sprint athletes.

Several studies observed significant correlations between force and sprint performance (Nesser et al., 1996), whereas others failed to claim such results (Kukolj et al., 1999; Marques and González-Badillo, 2006). Part of these discrepancies could be due to the fact that sprinting is a complex ability (Delecluse et al., 1995) that requires proper motor coordination between joints and muscles. Sprinting ability over very short distances (5 or 10 m) is considered by many researchers and practitioners to require specific strength qualities and running technique. It is generally accepted that shorter sprints require a greater contribution of concentric muscle contractions and knee extensor activity. Young et al. (1995) investigated the relationship between force measures (concentric only Smith squat jump with a 19 kg bar load from a 120º knee angle) and sprinting performance of 20 elite junior track and field athletes. The best predictors of starting performance (time to 2.5 meters) included force relative to body weight generated after 100 milliseconds from the start of the concentric jump movement (r = 0.73) and peak force (r = 0.72). Using a similar methodology, Wilson et al. (1995) were able to observe that force at 30 milliseconds in a concentric squat jump was significantly correlated to sprint performance (r = 0.62) and was able to effectively discriminate the good from the poor performers. The results of Wilson et al. (1995) and Young et al. (1995) also indicate that strength qualities such as the rate of force development or force applied at 100 milliseconds may be more important than maximal strength. However, the present study failed to show significant correlations not only between maximum rate of force development and both sprint times, but also between time to maximum rate of force and impulse with sprint distances. Moreover, the validity of isolating starting rate of force development has been corroborated by electromyographic studies and confirms the suggestion that RFD is, in part, determined by the innate qualities of the neuromuscular system, particularly the ratio of fast - to slow-twitch fibers in the muscles (Andersen and Aagaard, 2006; Vescovi and McGuigan, 2008). Young et al. (1995) has commented that RFD is regarded as a measure of very fast force production capabilities and found that the initial acceleration phase (0–2.5 m) is highly correlated (r = 0.86) with the force applied in a concentric - only squat jump. Therefore, specialization of the neuromuscular system to develop initial RFD is determined chiefly by the magnitude of external resistance (Andersen and Aagaard, 2006). On this, research has shown a correlation between RFD and initial acceleration, and the results of this study provide further evidence of the link between starting strength and improved acceleration in the early part of the sprint.

Given the impulse-momentum relationship, impulse is theoretically an important determinant of sprinting ability as indicated by biomechanics experts reporting the determinants of speed via qualitative models. This variable therefore should be of greater interest to the strength and conditioning community. However, impulse has received little attention from research on predictors of speed (González-Badillo and Marques, 2010). Wilson et al. (1995) investigated the relationship between impulse developed in the first 100 ms of a concentric Smith squat jump (unloaded) from 110° and 150° knee angles, and sprinting ability over 30 m. Although reported as non-significant, they reveal a moderate correlation (r = −0.49) between impulse at 150° and sprinting ability. Interestingly, the relationship between impulse at 110° and sprint ability was low (r = 0.06). Perhaps the influence of starting knee angle is critical to the relationship between concentric only machine squat-jump strength measures and sprint ability. It may be that the length-tension relationship of the hip and knee extensors at lower starting knee angles is biomechanically less specific to the actual knee angles encountered in 5 m sprints. It should be kept in mind that the sample used by other studies comprised subjects of different sports, levels and genders, which may account for the variation in results as compared to our study. Thus, a certain discrepancy should be expected between the CMJ mechanical impulse and sprint performance. Furthermore, sprint ability over short distances (<10 meters) is considered by many researchers and practitioners to require specific strength qualities and therefore training regimens (Vescovi and McGuigan, 2008; Chelly et al., 2009). It is generally considered that shorter sprints require greater contributions of concentric muscle contractions and knee extensor activity versus longer sprints that are characterized by greater stretch shortening cycle (SSC) and hip extensor activity. In addition to muscle-elastic mechanisms, the role of the stretch reflex has been related to enhancement of the SSC. According to Komi and Gollhofer (1997), an efficient SSC requires three basic conditions: well-timed muscle pre-activation (prior to the eccentric phase), short eccentric phase duration, and an immediate transition between eccentric and concentric phases. Furthermore, during muscle stretch, stretch induced reflex may play an important role in force generating coupling of cross-bridges due to reduced muscle stiffness.

The rate of force development (RFD) has been one of the most important variables to explain performance in activities where great acceleration is required (Moir et al., 2004; Vescovi and McGuigan, 2008; González-Badillo and Marques, 2010). This can be related to the fact that the greater the RFD, the higher will be the power and the force generated against the same load. In most sports activities, the RFD is strongly related to performance abilities such as sprinting, in which force production time is very small. Unfortunately, previously published reports examining the relationship between the rate of force development and sprint performance have provided equivocal findings, with some studies reporting a significant relationship and others failing to observe a positive association (Moir et al., 2004). The present study failed to indicate a significant association between different rates of force measurements and 5 meter sprint time. It is difficult to compare the results of these studies because they markedly differ in a number of factors, including the method of measurement. Yet, the variations in correlation coefficients may have been explained by the differences in reliability for measuring peak of rate of force development (CV= 6 to 14%) when compared to measuring peak force (CV=4 to 8%).

The need for strength and power requirements in athletes is sport specific. Individual sports, such as track and field, often have very specific strength and power profiles with predetermined requirements allowing a more simplistic prescription of training requirements by the coach. For example, the acceleration phase and predominantly the initial acceleration phase (0–10 m) are of major importance to athletes. Research on track sprinters starting from blocks has identified that the first few ground contact phases of a short sprint are dominated by propulsive forces when compared to braking forces (Mero, 1988), and by concentric muscle actions (Chelly et al., 2009). The average horizontal impulse of track sprinters in the blocks and during the propulsive phase of the first ground contact have also shown significant correlations with initial running velocity when they are expressed relative to body weight (Mero et al., 1983; Mero, 1988). These findings emphasize the dominance of the propulsive phase during initial acceleration, and the importance of propulsive force developed during the first few foot contacts of the sprint in maximizing initial running velocity. As running velocity approaches maximum, those strength measures that require force to be produced at high velocities have been reported to be significantly related to sprint performance (Young et al., 1995; Nesser et al., 1996).

According to González-Badillo and Marques (2010), there is a substantial body of literature focused on clarifying the relationship between mechanical power output and athletic performance. A concern that was raised by Harris et al. (2008) is that the power measurements and protocols used in these studies can vary considerably. Along the same line, Carlock et al. (2004) stated that making comparisons between various studies is rather difficult because there are different exercises being used to measure peak power output. Despite these limitations, there is a growing body of literature on the relationship of power to sprint performance. A majority of researchers have found moderate to strong correlations between jump height (and/or relative peak power), measured during a vertical jump, and sprinting performance (Cronin and Hansen, 2005; Harris et al., 2008; Habibi et al., 2010). Theoretically, there should be a significant relationship between these parameters, as a rapid SSC occurs both in jumping and sprinting. The present study indicated that power could explain approximately 36% of the sprint performance. Sleivert and Taingahue (2004) who investigated the relationship between 5 m sprint times and power variables in trained athletes could observe that both mean power and peak power relative to body mass were strongly negatively correlated with 5 meter sprint time (r = − 0.64 to 0.68). The authors chose not to incorporate body mass (so-called system mass) into the equation of force, asserting that it is not strictly mechanically correct to do so. Sleivert and Taingahue (2004) noted that not using system mass has the effect of markedly reducing power outputs and altering the point on the power. Cronin and Hansen (2005) noticed that peak power output measured on a force platform in the squat jump (expressed relative to subject’s body mass) was determined to be related to the 5 (r = − 0.55, p <0.05) and 10 m sprint (r = − 0.54, p <0.05) times.

When the power movements are in the vertical plane (e.g., vertical jump), force calculations must be adjusted to include the effects of gravity on the load, and this has the effect of increasing the relative load at which peak power occurs (Carlock et al., 2004). Furthermore, some studies have also incorporated body mass into their power calculations when exercises are performed in the vertical plane, on the assumption that it is also being accelerated (Markovic et al. 2004). This has the effect of markedly increasing absolute power and concomitantly reducing the relative load at which peak power occurs. Considering that not all of the body mass is usually accelerated and that the velocity of the bar (not the center of mass) has been measured and used in these calculations, many of these power equations are not strictly mechanically correct. Clearly, a standard method for calculating power in resistance training movements needs to be agreed upon. In the meantime, researchers and practitioners should be aware of the implications resulting from including or excluding body mass in power calculations for exercises occurring in the vertical plane.

This study presented some limitations that should be considered. First, this study used a sample of trained participants but not elite athletes, which may have an influence on correlations if outliers are present. Normality was assessed for each of the performance outcomes and it seems the results of this investigation were not affected by outliers. Second, we only assessed lower body kinetics and not other kinetic and kinematic variables playing an important role in short sprint performance. Given the fact that sprinting is a highly complex motor skill, it would be unlikely to find a single test that accounts for nearly all of the variability in sprinting.

As a conclusion, one can state that within the confines of our study limitations, these findings highlight the important relationship between 5 m sprint and maximal lower body strength, as assessed by the force, power and bar velocity/displacement. These findings should be interpreted with caution since correlations provide only associations and do not represent causation.

## Figures and Tables

**Table 1:** *Correlations between 5 m sprint performance and strength metrics of the CMJ using a linear transducer*.

**Variables**	***r values***
	
Bar displacement (m)	− 0.682**
Bar displacement time duration (ms)	− 0.699**
Propulsive time duration (ms)	− 0.737**
Time to peak bar velocity (ms)	− 0.664**
Mean bar velocity (m/s)	0.231 ns
Peak bar velocity (m/s)	0.308 ns

Mean force (N)	0.377 ns
Mean force until peak velocity (N)	0.680**
Mean propulsive force (N)	0.801**
Peak force (N)	0.431 ns
Time to peak force (ms)	− 0.127 ns
Mechanical impulse (N.s)	− 0.698**
RFD_max_. (N x s^−1^)	0.354 ns
Time to RFD_max_. (ms)	0.066 ns

Mean power (W)	0.233 ns
Mean power until peak velocity (W)	0.648**
Mean propulsive power (W)	0.715**
Peak power (W)	0.500 ns
Time to peak power (m/s)	− 0.660**

Significance:

** p<0.01; ns: non-significant
